# The melatonin metabolite N1‐acetyl‐5‐methoxykynuramine facilitates long‐term object memory in young and aging mice

**DOI:** 10.1111/jpi.12703

**Published:** 2020-11-20

**Authors:** Hikaru Iwashita, Yukihisa Matsumoto, Yusuke Maruyama, Kazuki Watanabe, Atsuhiko Chiba, Atsuhiko Hattori

**Affiliations:** ^1^ Department of Biology College of Liberal Arts and Sciences Tokyo Medical and Dental University Chiba Japan; ^2^ Department of Materials and Life Sciences Faculty of Science and Technology Sophia University Tokyo Japan

**Keywords:** aging, long‐term memory, melatonin, N1‐acetly‐5‐methoxykynuramine, N1‐acetyl‐N2‐formyl‐5‐methoxykynuramine, novel object recognition, short‐term memory

## Abstract

Melatonin (MEL) has been reported to enhance cognitive processes, making it a potential treatment for cognitive decline. However, the role of MEL’s metabolites, N1‐acetyl‐N2‐formyl‐5‐methoxykynuramine (AFMK) and N1‐acetyl‐5‐methoxykynuramine (AMK), in these effects are unknown. The current study directly investigated the acute effects of systemic MEL, AFMK, and AMK on novel object recognition. We also analyzed MEL, AFMK, and AMK levels in hippocampus and temporal lobe containing the perirhinal cortex following systemic MEL and AMK treatment. AMK administered post‐training had a more potent effect on object memory than MEL and AFMK. AMK was also able to rescue age‐associated declines in memory impairments when object memory was tested up to 4 days following training. Results from administering AMK at varying times around the training trial and the metabolism time course in brain tissue suggest that AMK’s memory‐enhancing effects reflect memory consolidation. Furthermore, inhibiting the MEL‐to‐AMK metabolic pathway disrupted object memory at 24 hours post‐training, suggesting that endogenous AMK might play an important role in long‐term memory formation. This is the first study to report that AMK facilitates long‐term object memory performance in mice, and that MEL crosses the blood‐brain barrier and is immediately converted to AMK in brain tissue. Overall, these results support AMK as a potential therapeutic agent to improve or prevent memory decline.

## 1 INTRODUCTION

The percent of middle‐aged and elderly individuals with varying degrees of cognitive decline and risk of developing neurodegenerative disorders, such as Alzheimer's disease (AD), is persistently increasing.^1^ Mild cognitive impairment (MCI) is an etiologically heterogeneous syndrome characterized by cognitive impairment that precedes dementia.^2^ Approximately 12% of MCI patients develop AD or other dementia disorders every year,^2^ demonstrating an urgency to develop drugs that improve MCI or prevent its transition to dementia.

Melatonin (MEL) is primarily produced and secreted by the pineal gland. It is known for its key regulatory role in circadian rhythms and may also regulate memory formation.^3^, ^4^ The effects of long‐term MEL paired with free‐radical scavenger treatment have been evaluated regarding learning and memory.^5^ However, MEL’s acute effects on learning and memory remain unclear.

MEL is converted to N1‐acetyl‐N2‐formyl‐5‐methoxykynuramine (AFMK) and then to N1‐acetyl‐5‐methoxykynuramine (AMK) in the brain.^6^, ^7^, ^8^ MEL and AMK may interact with calmodulin (CaM),^8^ which plays a crucial role in various neural functions, including cognition.^9^, ^10^ These findings suggest that MEL and its metabolites may affect cognitive functions. Notably, AMK has a higher affinity for CaM than MEL,^8^ indicating that MEL’s metabolites may show superior cognitive effects. However, we are unaware of any studies reporting the effects of AFMK or AMK on learning and memory.

The current study examined the acute effects of intraperitoneal (i.p.) MEL, AFMK, and AMK injections on learning and memory performance using the novel object recognition (NOR) test. The NOR test is a rodent model of pure declarative memory, in which memory is quantified based on the natural tendency of animals to explore novel objects.^11^, ^12^ Because we found the AMK facilitated NOR performance in young mouse, we investigated whether AMK could improve NOR performance in middle‐aged and old mice with age‐associated cognitive deficits. The role of endogenous AMK in learning and memory was evaluated by blocking the metabolic pathway in which MEL is converted into AMK. The present data indicate that AMK deserves further consideration as a therapeutic agent for memory decline in middle‐aged and elderly populations.

## 2 METHODS

### 2.1 Animals

Eight‐week‐old male Scl:ICR mice, a MEL‐deficient strain^13^ (Table S1), were purchased from Sankyo Labo Service Corporation Inc (Tokyo, Japan) and individually housed with food and water ad libitum at 22°C under a 12‐hour light/12‐hour dark cycle. Mice were assigned to experiments when they were 2 months (young), 14 months (middle‐aged), or 21 months (old) of age. Experiments were conducted during the latter half of the 12‐h light period. All experimental procedures were approved by the Institutional Animal Experiment Committee at Sophia University and were designed to minimalize pain or discomfort.

### 2.2 Drugs and treatments

MEL (Sigma‐Aldrich, St. Louis, MO, USA), AFMK (Cayman Chemical, Ann Arbor, MI, USA), AMK (Toronto Research Chemicals, Toronto, Canada), 6‐hydroxymelatonin (6OHMEL; Sigma‐Aldrich), and isoflurane (Pfizer, Tokyo, Japan) were purchased for this study. Ethanol (EtOH) and dimethyl sulfoxide (DMSO) were supplied by Kanto Chemical (Tokyo, Japan). Acetone, acetic acid, ammonium acetate, and methanol (MeOH) were supplied by Wako (Tokyo, Japan). Milli‐Q water was used in all assays, and all reagents were of analytical grade or higher.

Animals were intraperitoneally treated with MEL (1.0 or 5.0 mg/kg in saline containing 1% EtOH), AFMK (0.1 or 1.0 mg/kg in saline), AMK (0.01, 0.1, or 1.0 mg/kg in saline), or 6OHMEL (1.0 mg/kg in saline containing 1% DMSO) as early as possible before the dark period. To assess inhibition of the MEL‐to‐AMK metabolic pathway, norharmane (Wako Pure Chemical Industries, Osaka, Japan), an indoleamine 2,3‐dioxygenase (IDO) inhibitor, was injected 5 hours before the training phase.

### 2.3 MEL, AFMK, and AMK assays

#### 2.3.1 Sample preparation

Mice were deeply anesthetized with isoflurane 5, 15, 30, or 60 minutes after i.p. injection of 1.0 mg/kg MEL (n = 4) or 5, 15, 30, 60, 120, or 240 minutes after i.p. injection of 1.0 mg/kg AMK (n = 6‐10). The mice were decapitated and the hippocampus (HP) and a portion of the temporal lobe containing the perirhinal cortex (TL), which are known as brain regions involved on cognitive memory, were collected. HP and TL tissues were also collected from untreated mice to measure basal MEL, AFMK, and AMK levels. All sample tissues were immediately frozen in liquid nitrogen and stored at −80°C prior to the assays.

Sample tissues were added to 100 µL of Milli‐Q water and were homogenized on ice. Subsequently, four volumes of acetone were added and the samples were vortexed for 5 minutes, followed by centrifugation at 18,000*g* for 10 minutes. Supernatants were then transferred into clean test tubes and were evaporated to dryness at 65°C under a stream of nitrogen gas. After dissolving residues into 100 µL of Milli‐Q water, the mixtures were passed through filters with 0.22‐μm pores (Centrifugal Filter Units Ultrafree‐MC‐GV; Merck Millipore, Guyancourt, France) and were stored at −80°C until liquid chromatography‐tandem mass spectrometry (LC‐MS/MS) analyses.

#### 2.3.2 Liquid chromatography and mass spectrometric conditions

MEL, AFMK, and AMK were simultaneously analyzed in tissues using a previously described LC‐MS/MS protocol.^14^ Briefly, 10‐µL samples were injected into a high‐performance liquid chromatography system (AC30AD; Shimadzu Corporation, Kyoto, Japan) equipped with a C18 2.0 × 150‐mm, 3‐µm Kinetex column (Tosoh, Japan). The mobile phase utilized 10 µmol/L ammonium acetate in 0.05% (v/v) acetic acid with varying concentrations of MeOH. The linear gradient was run over 20 minutes from 5% to 50% MeOH maintained thereafter in 100% MeOH for 10 minutes. The flow rate was 0.3 mL/min and the autosampler and column oven were maintained at 4°C and 25°C, respectively. MEL, AFMK, and AMK were detected using a triple quadrupole mass spectrometer (LCMS‐8050; Shimadzu) and were quantified using multiple reaction monitoring with the transition of parent ions to product ions. The transitions were *m*/*z* 233.0‐130.0, *m*/*z* 265.0‐136.1, and *m*/*z* 237.0‐136.1 for MEL, AFMK, and AMK, respectively. The extraction efficiencies were 94.35% ± 2.64%, 92.73% ± 1.13%, and 85.03% ± 0.91% for MEL, AFMK, and AMK, respectively. The limits of sensitivity for a 2:1 signal‐to‐noise ratio were 11.5, 15.5, and 18.1 fg for MEL, AFMK, and AMK, respectively. The intra‐assay variation coefficients were 3.94%, 3.41%, and 7.44% for MEL, AFMK, and AMK, respectively.

### 2.4 NOR test

NOR was conducted in an open‐field polypropylene arena (40 cm width × 30 cm depth × 30 cm height). The floor was divided into 20 equal sections by drawing grid lines. The objects were ceramic dolls and stainless cruets covered with steel, which are too heavy to be displaced by animals.

Animals were first individually habituated to the empty arena for 5 minutes daily for 3 days. All experiments used 1 minute training trials, except for the norharmane experiment. In the training phase, animals performed 1‐5 acquisition trials (one 1 minute‐ or five 1 minute trials) where they were allowed to freely explore two identical objects symmetrically placed in the arena at a distance of 5 cm from the wall and 15 cm from each other. Spaced training is reportedly superior to massed training for LTM formation.^15^, ^16^ Therefore, we trained animals using five 1 minute acquisition trials with a 1 hour inter‐trial interval. Mice were subjected to the test phase after 15 minutes to 24 hours delay. One of the familiar objects was replaced with a novel object at the same location and the mice were given 5 minutes to freely explore. Object novelty and location were counterbalanced within experimental groups to eliminate potential biases caused by preference for particular objects or locations. The objects were thoroughly cleaned with 70% EtOH after each trial to minimize the presence of olfactory trails. Mice were placed in the middle of the two objects facing the wall at the beginning of the trial and were recorded using a video camera mounted above the arena.

The time spent exploring the objects was quantified by a blinded trained observer using the recordings. Exploratory behavior was defined as the animal directing its nose toward the object at a distance of <1 cm. Animals were excluded from data analyses if they met the following criteria: (a) spent 2 seconds or less exploring objects during any acquisition trial; (b) spent 10 seconds or less exploring objects during the test trial; (c) did not explore both objects during the training and test phases. The discrimination index (DI) was calculated as follows: percent of time spent exploring the novel object divided by the total time spent exploring both objects during the test phase. Object recognition is considered as DIs that are significantly above chance performance (50%).^11^, ^17^ Off‐target drug effects were quantified using spontaneous locomotor activity, defined as the number of times mice crossed the grid lines with all four paws (grid‐crossing), and the total time spent exploring both objects during each phase.

### 2.5 Statistical analyses

All data are provided as the mean ± standard error of the mean. One‐sample *t* tests were used to assess whether DIs significantly differed from 50%. Prior to analysis of variance (ANOVA), data sets were checked for normality and homogeneity of variances by Kolmogorov‐Smirnov and Levene's tests, respectively. Groups comparisons were performed using one‐way ANOVA or Kruskal‐Wallis tests with Tukey or Steel‐Dwass post hoc tests, as appropriate. Differences were considered significant when *P* ≤ .05.

## 3 RESULTS

### 3.1 Short‐term memory (STM) and LTM NOR performance in untreated mice

**FIGURE 1:**
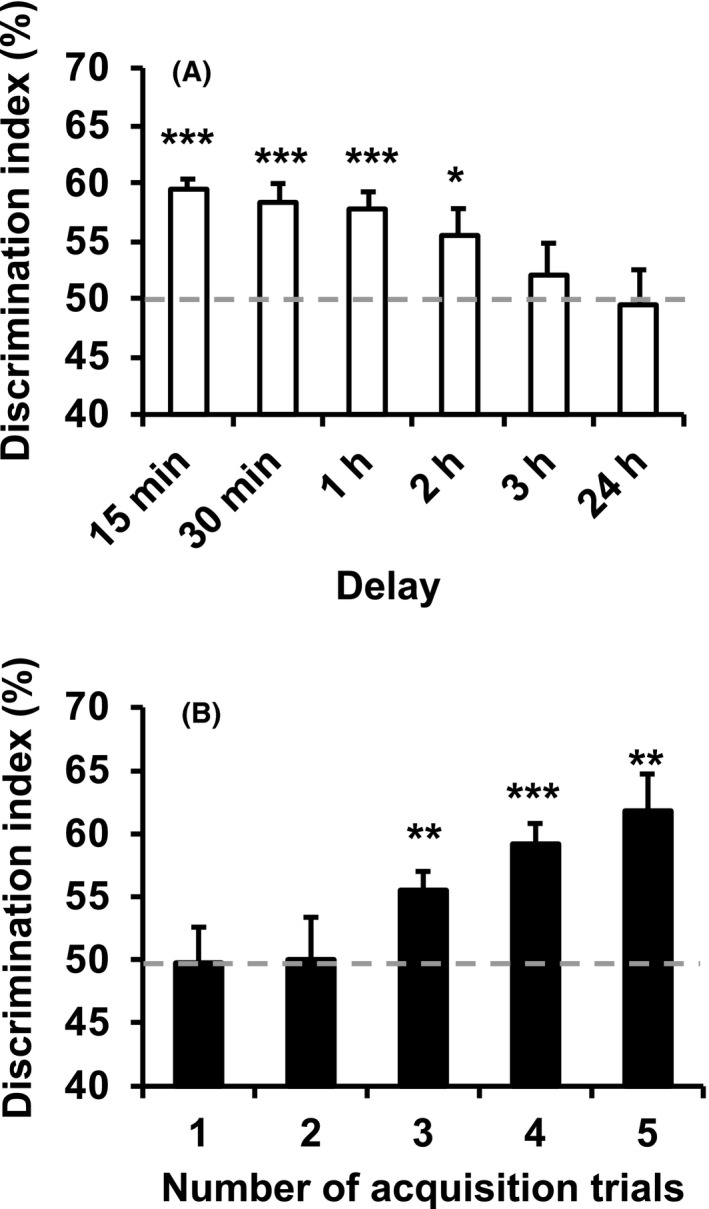
Assessment of short‐ and long‐term object memory in untreated mice. A, Increasing delays between a single 1‐minute training trial and the test trial reduced object memory. B, Increasing the number of 1‐minute training trials with a 1‐hour inter‐trial interval increased object memory 24 hours post‐training. Data are presented as the mean ± standard error. *P < .05, **P < .01, and ***P < .001 indicate significantly different than chance performance (50%). Discrimination index (%) = time exploring novel object/ total object exploration time during test X 100

A single 1‐minute acquisition trial produced DIs that were significantly greater than those of chance performance when tested at 15 minutes, 30 minutes, 1 hour, and 2 hours (all *P* < .05) later after the acquisition trial, but not 3 hours and 24 hours later (all *P* > .05) (Figure 1A). A one‐way ANOVA revealed that the inter‐trial interval significantly influenced the DI (*P* < .05). These data indicate that mice were able to retain a STM for 2 hours in the present experimental design.

Further experiments tested NOR 24 hours following 1‐5 training trials (Figure 1B). The DIs were significantly different from those of chance performance following three or more acquisition trials (all *P* < .01), indicating that repeated training allowed mice to establish a LTM. A one‐way ANOVA confirmed that the number of training trials influenced the DI *(P* < .05).

### 3.2 Effects of MEL, AFMK, and AMK on LTM performance

**FIGURE 2:**
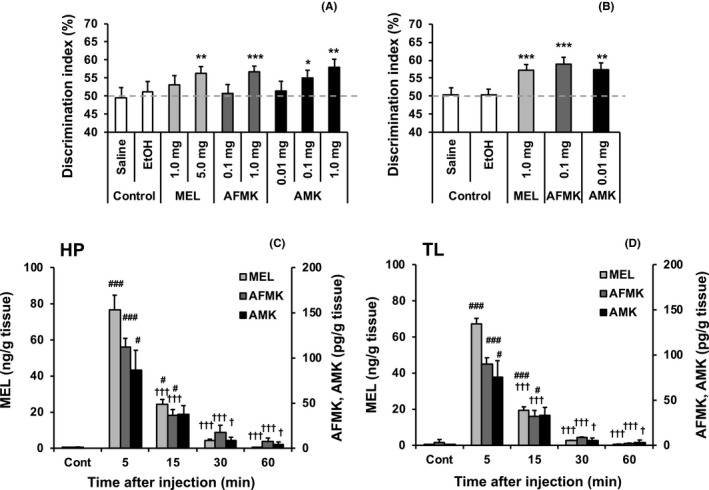
Acute melatonin, AFMK, or AMK effects on long‐term object memory and melatonin metabolism in brain tissue. A, Vehicle (Control), melatonin (MEL), N1‐acetyl‐N2‐formyl‐5‐methoxykynuramine (AFMK), or N1‐acetyl‐5‐methoxykynuramine (AMK) were systemically administered 1 hour following a single 1‐minute training trial. All drugs increased object recognition at 24 hours post‐training. B, Systemic MEL, AFMK, or AMK administration 1 hour following two 1‐minute training trials increased object memory at 24 hours post‐training. C, D, MEL, AFMK, and AMK concentrations in the hippocampus (HP; C) and temporal lobe containing the perirhinal cortex (TL; D) were measured in untreated control mice (Cont) or after systemic treatment with 1.0 mg/kg MEL. Data are presented as mean ± standard error. A and B, *P < .05, **P < .01, and ***P < .001 indicate significantly different than chance performance (50%). C and D, ^#^ *P* < .05 and ^###^ *P* < .001 indicate significantly different than Cont. ^†^ *P* < .05 and ^†††^ *P* < .001 indicate significantly decreased compared to the respective 5‐minutes value. Discrimination index (%) = time exploring novel object/ total object exploration time during test X 100

Drug effects were assessed using a single training session and a 24‐hours inter‐trial interval. Varying doses of MEL, AFMK, or AMK were administered 60 minutes after the training phase. Although the low drug doses did not affect the DI (all *P* > .05), the higher doses of all drugs significantly increased the DI compared with chance performance (all *P* < .05) (Figure 2A), indicating facilitated LTM. AMK required the lowest dose to enhance LTM, indicating that among all substances tested this one is the most potent. When training was increased to two trials, previously sub‐effective doses of MEL (1.0 mg/kg), AFMK (0.1 mg/kg), and AMK (0.01 mg/kg) significantly increased the DI compared with chance performance (all *P* < .01) (Figure 2B). These data demonstrate that MEL, AFMK, and AMK reduced the minimum training requirement to establish a LTM.

A separate experiment examined the acute effects of 6OHMEL, a MEL metabolite that is found mainly in the liver. The drug was administered 60 minutes following two training trials and NOR was tested 24 hours later. MEL (1.0 mg/kg) significantly facilitated LTM (*P* < .001), whereas 6OHMEL (1.0 mg/kg) had no effect (*P* > .05) (Figure S1), indicating that these effects may be specific to MEL metabolism to AMK in the brain.

### 3.3 MEL, AFMK, and AMK concentrations in HP and TL tissues following MEL treatment

MEL (1 mg/kg) increased MEL, AFMK, and AMK concentrations across time in HP (one‐way ANOVA, *P* < .001) and TL tissues (one‐way ANOVA, *P* < .001) (Figure 2C and D). MEL, AFMK, and AMK levels in both regions were significantly increased compared with those in basal control samples at 5 minutes following MEL administration (all *P* < .05) (Figure 2C and D), indicating that MEL crosses the blood‐brain barrier and is immediately converted to AFMK and then AMK. All compound concentrations were highest 5 minutes following treatment (Figure 2C and D), indicating that they rapidly reached peak levels and were then metabolized and cleared (Figure 2C and D).

### 3.4 AMK’s LTM effects depend on the time of administration

**FIGURE 3:**
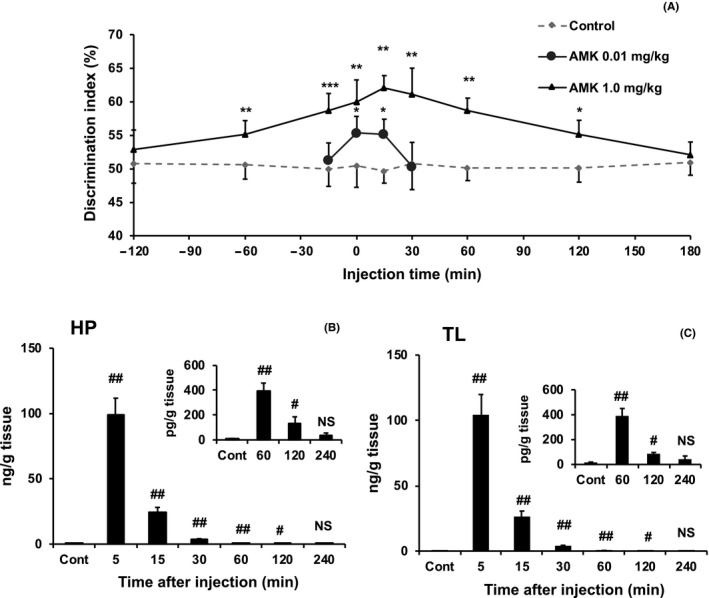
Time course of long‐term object memory and AMK levels in brain tissue by acute AMK administration. A, Systemic N1‐acetyl‐5‐methoxykynuramine (AMK) administration at nine time points before or after a single 1‐minute training trial (0 time) enhanced object memory at 24 hours post‐training. B and C, AMK levels in hippocampus (HP; B) and temporal lobe containing the perirhinal cortex (TL; C) were measured in untreated control mice (Cont) or after systemic treatment with 1.0 mg/kg AMK. To confirm the values from 60 minutes to 240 minutes after AMK injection, an inset at the top right of the figure was inserted. Data are presented as mean ± standard error. A, *P < .05, **P < .01, and ***P < .001 indicate significantly different than chance performance (50%). B and C, ^#^ *P* < .05 and ^##^ *P* < .01 indicate significantly different than Cont. Discrimination index (%) = time exploring novel object/ total object exploration time during test X 100

Mice were treated with vehicle, 0.01 mg/kg, or 1.0 mg/kg AMK ranging from 120 minutes before to 180 minutes after a single training trial and tested 24 hours later. Vehicle administered across the time points did not significantly alter the DI (Figure 3A). Administration of 1.0 mg/kg AMK from 60 minutes before to 120 minutes after training significantly increased the DI compared with chance performance (all *P* < .05), whereas 0.01 mg/kg AMK only increased the DI when administered 0 and 15 minutes following training (all *P* < .05). These results indicate that AMK can facilitate LTM when administered over a broad range of time, but these effects are dose‐dependent and lower doses are more sensitive to temporal effects.

No significant effects of 1.0 mg/kg AMK were observed on total exploration time or grid‐crossing events at 15, 60, or 120 minutes following drug administration (all *P* > .05) (Figure S2A and B).

### 3.5 AMK concentration in HP and TL tissues following AMK treatment

AMK (1 mg/kg) treatment increased AMK levels in the HP (Kruskal‐Wallis, *P* < .001; Figure 3B) and TL (Kruskal‐Wallis, *P* < .001; Figure 3C). Steel‐Dwass post hoc analyses revealed that AMK concentrations were significantly increased compared with those in the untreated controls at 5, 15, 30, 60, and 120 minutes after administration in both regions (all *P* < .05), peaking at the 5‐minutes time point. These results confirm that AMK easily passes the blood‐brain barrier and is rapidly metabolized and cleared.

### 3.6 AMK effects on LTM across age

**FIGURE 4:**
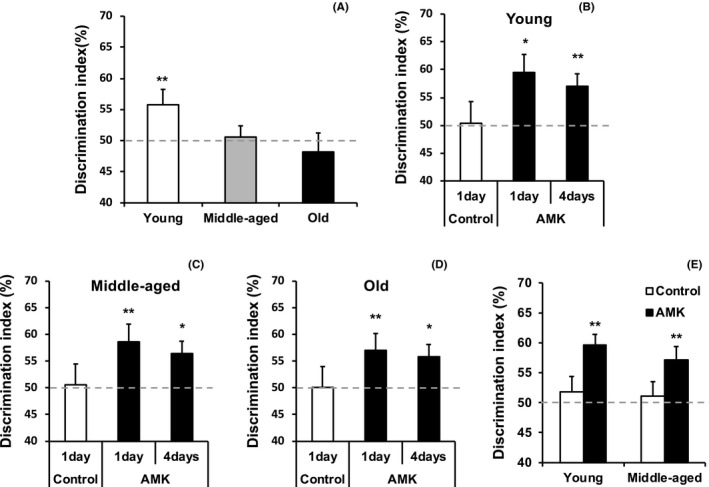
Acute AMK effects on long‐term object memory in young, middle‐aged, and old mice. A, Three 1‐minute training trials revealed age‐associated object memory decline in middle‐aged and old mice at 24 hours post‐training. B‐D, Systemic N1‐acetyl‐5‐methoxykynuramine (AMK; 1 mg/kg) administered after a single 1‐minute training trial enhanced object memory at 1 and 4 days post‐training in all age groups. E, A reduced systemic AMK dose (0.1 mg/kg) administered after two 1‐minute training trials significantly increased object memory at 24 hours post‐training in young and middle‐aged mice. Data are presented as mean ± standard error. *P < .05 and **P < .01 indicate significantly different than chance performance (50%). Discrimination index (%) = time exploring novel object/ total object exploration time during test X 100

Following three training trials, the DI in young mice, but not that of middle‐aged or old mice, was significantly greater than chance performance levels at 24 hours post‐training (Figure 4A). This indicates that NOR in mice declines by 14 months of age.

To observe the effects of AMK on age‐related memory, mice were exposed to one training session, received 1.0 mg/kg AMK 15 minutes post‐training, and were tested for NOR 1 or 4 days after training. This protocol did not produce significant DIs in control mice of any age at 1 day post‐training, but AMK‐treated mice showed significant DIs 1 or 4 days after training, regardless of the age (all *P* < .05) (Figure 4B–D). Additionally, 0.1 mg/kg AMK administered 15 minutes after two training trials significantly increased the DIs 1 day post‐training in young and middle‐aged mice (all *P* < .01) (Figure 4E). These results indicate that AMK can significantly facilitate extended LTM across all age groups.

### 3.7 Effect of inhibiting the MEL‐to‐AMK metabolic pathway on LTM performance

To assess how the MEL‐to‐AMK metabolic pathway regulates LTM, we administered the IDO inhibitor norharmane 5 hours before a single 5‐minutes training trial. The training trial was immediately followed by administration of vehicle, 0.01 mg/kg MEL, or 0.001 mg/kg AMK, the minimum effective doses to produce LTM when the NOR test was performed 24 hours post‐training (Figure S3).

**FIGURE 5:**
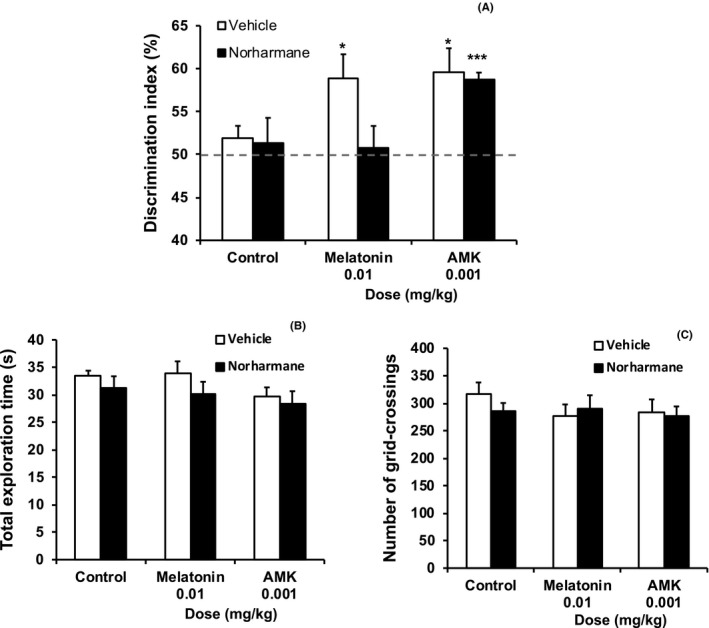
Inhibiting melatonin to AMK metabolism alters long‐term object memory. A, Norharmane or vehicle was systemically administered 5 hours before a single 5‐minutes training trial and immediately followed by administration of vehicle (Control), melatonin (MEL), or N1‐acetyl‐5‐methoxykynuramine (AMK). Norharmane blocked the object memory enhancement effect of MEL, but not that of AMK, at 24 hours post‐training. B and C, Total exploration time (B) and grid‐crossing events (C) during the 24 hours post‐training phase were not affected by drug treatment. Data are presented as mean ± standard error. *P < .05 and ***P < .001 indicate significantly different compared to chance performance (50%). Discrimination index (%) = time exploring novel object/ total object exploration time during test X 100

At 24 hours post‐training, MEL and AMK alone significantly increased the DI compared with that of chance performance (all *P* < .05) (Figure 5A). However, norharmane pretreatment blocked the effect of MEL (*P* > .05), but not that of AMK (*P* < .001), on the DI (Figure 5A). Notably, norharmane treatment did not affect total exploration time or grid‐crossing effects in the 24 hours post‐training phase (Figure 5B and C). These data indicate that interfering with MEL‐to‐AMK metabolism by inhibiting IDO blocked enhanced LTM.

## 4 DISCUSSION

**FIGURE 6:**
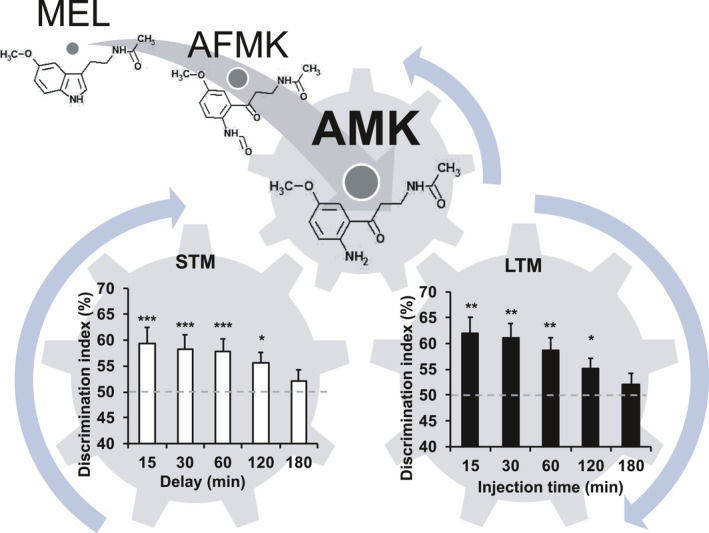
Proposed role of short‐ to long‐term memory transfer by endogenous AMK. Melatonin (MEL) and its metabolites improve long‐term memory (LTM) formation by facilitating the transfer of short‐term memories (STMs) to LTMs, with N1‐acetyl‐5‐methoxykynuramine (AMK) primarily responsible for these effects. The STM graph was adapted from Figure 1A to show object recognition over multiple delays following a single 1‐minute training trial. The LTM graph was modified from Figure 3A to show the effects of AMK administration at 15‐180 minutes after a single 1‐minute training trial on object memory at 24 hours post‐training. The AMK treatment window that facilitated LTM overlapped with the period where an STM is retained—120 minutes. Data are presented as mean ± standard error. *P < .05, **P < .01, and ***P < .001 indicate significantly different than chance performance (50%). Discrimination index (%) = time exploring novel object/ total object exploration time during test X 100

A single treatment with MEL or its metabolites, AFMK and AMK, remarkably facilitated LTM in young mice. Because MEL, AFMK, and AMK were administered after training, their effects on LTM likely reflect processes that follow memory encoding. Given the short half‐lives of these agents in brain tissue, their effects are also unlikely to reflect altered memory retrieval. Thus, the observed memory‐enhancing effects are likely mediated by memory consolidation, when a STM becomes a LTM (see Figure 6). MEL was immediately converted to AFMK and then to AMK in brain tissue following i.p. administration. Treatment with an IDO inhibitor, which inhibits MEL‐to‐AMK metabolism, blocked the enhanced LTM observed when MEL was administered alone. These data suggest that MEL’s effects on the memory consolidation process may depend on its conversion to AMK.

As the most potent LTM facilitating compound, our next set of experiments focused on the role of AMK in memory consolidation and encoding. AMK administered 60 minutes before to 120 minutes after training enhanced LTM, indicating its broad temporal effects on LTM at a 1 mg/kg dose. The 0.01 mg/kg AMK dose enhanced memory only when given 0 and 15 minutes following training. This may reflect AMK levels in brain tissue, which remain elevated but rapidly decline over an extended period following 1.0 mg/kg AMK treatment. The low dose of AMK may minimally elevate AMK in brain tissue. These data suggest that AMK facilitates LTM by acting on memory consolidation rather than memory encoding, and the effects of early treatment with the high dose can be attributed to elevated AMK levels that could sufficiently facilitate LTM consolidation processes during and shortly after the training phase. AMK administered 180 minutes following training did not enhance LTM, which was consistent with the inter‐trial interval where untreated control mice were unable to retain a STM after a single acquisition trial (see Figure 6). These results suggest that AMK has no beneficial effects on memory consolidation more than 2 hours after training because no STM remains available for conversion into a LTM.

Acute MEL treatments can impair or improve cognitive performance. Positive MEL effects have mainly been observed in learning studies that take advantage of spontaneous animal behaviors. A single MEL administration facilitated social memory in an olfactory memory task.^18^ In the NOR task, a single MEL administration before training trials facilitated LTM assessed 24 hours post‐training,^19^ which is consistent with the current findings. A single injection of melatonin is expected to increase melatonin levels in SCN as well as the hippocampus. MEL administration may cause phase‐shift in SCN, which in turn may affect learning and memory formation. We conducted an experiment in which ICR mice were housed under a 12‐hour light/12‐hour dark (12L12D) cycle, the same conditions used in the learning and memory tests. Melatonin was also administered once at the same time point used in the learning and memory experiment. We recorded and compared the acrophase 3 days before and 3 days after the administration of melatonin (Figure S4). The average phase delay was 26.4 minutes in the control group and 12.0 minutes in the melatonin‐administered group, with no significant difference between groups. Similarly, acrophase data acquired 2 days before and 2 days after the administration of melatonin did not yield significant difference between groups. Actograms have been previously used to investigate the occurrence of phase‐shift due to melatonin administration under constant darkness conditions.^20^, ^21^ Our data, however, suggest that the effect of phase‐shift is less likely to occur under the 12L12D cycle than under the constant darkness condition. Furthermore, it has been demonstrated that a single MEL administration facilitates cognitive performances in humans.^22^ Together, these findings suggest that MEL treatments acutely enhance learning and memory in learning tasks without reinforcing or punishing stimuli. MEL treatment is likely to be effective for human memory as well, since human memory that occurs daily does not generally depend on its association with reinforcing or punishing stimuli.

Conversely, negative MEL effects have mainly been demonstrated in studies using operant tasks with reinforcing or punishing stimuli. Acute MEL treatment impaired memory encoding, but not retrieval, in an active avoidance task adapted for zebrafish.^23^ Rodent studies examining punishing stimuli in passive avoidance^24^ or fear conditioning^25^ tasks have shown that a single MEL administration before learning trials impairs learning and memory performance. A single MEL administration before the learning trials for an 8‐arm radial maze task with reinforcing stimuli also induced learning and memory impairments.^26^ Furthermore, the amygdala mediates emotional memories^27^ involved in these learning tasks. Intra‐amygdala microinjection of MEL before learning trials impaired learning and memory performance in a water maze task.^28^ MEL has sedative, anxiolytic, and analgesic activities^29^ that may indirectly induce negative effects on the cognitive performance required for these learning tasks.

MEL is metabolized to AFMK by IDO^7^, ^30^ or free‐radical scavenging,^30^, ^31^ then AFMK is metabolized to AMK by arylamine formamidase or catalase pathway.^32^ The AMK’s physiological role has not been well investigated. To the best of our knowledge, AMK is only known to interact with CaM and has a higher CaM affinity than MEL.^8^ CaM plays an integral role in synaptic plasticity. Stimulation of Ca^2+^/CaM‐dependent protein kinase (CaMK) II via Ca^2+^ influx through N‐methyl‐D‐aspartate–type glutamate receptors is one of the core signaling mechanisms that induces early long‐term potentiation (LTP).^33^ CaMKIV pathway activation results in cAMP‐response element binding protein (CREB) phosphorylation at Ser133^9^, ^10^ followed by activation of CREB‐dependent gene expression, a crucial step in the molecular cascade that mediates LTM formation.^9^, ^10^ A previous study showed that selectively expressing a dominant Ca^2+^/CaM inhibitor in forebrain neuron nuclei diminished neuronal activity‐induced CREB phosphorylation, reduced activity‐induced gene expression, impaired hippocampal LTP, and severely impaired LTM, but not STM, formation.^34^ Therefore, AMK’s actions on LTM may be related to the CaM‐dependent signaling pathway. Although the mechanisms by which AMK facilitated LTM are currently unclear, investigating AMK’s effects on synaptic plasticity and related signaling pathways may help to elucidate the mechanisms underlying AMK’s ability to facilitate LTM.

Previous studies evaluating chronic MEL treatment have indicated predominantly beneficial effects on cognitive deficits in AD^35^ and in various animal models such as AD, Down syndrome, sleep deprivation, and chemically induced memory impairments.^5^ It has been suggested that MEL has anti‐inflammatory, anti‐amyloidogenic, and antioxidant properties^5^, ^36^ that underlie its potential to reduce brain damage and improve learning and memory deficits over long‐term treatment.^5^ These effects may also depend on MEL’s metabolites. In the present study, a single AMK administration enhanced LTM, even in mice with age‐associated memory decline.^37^, ^38^ This suggests that AMK is a potential therapeutic agent to improve quality of life in individuals with MCI.

In conclusion, we provide the first evidence that acute treatment with AMK, a MEL metabolite, facilitates LTM with superior effects compared to MEL. The present data strongly suggest that AMK plays a key role in facilitating STM‐to‐LTM transfer of information (Figure 6).

## CONFLICT OF INTEREST

The authors declare no competing financial interests.

## AUTHOR CONTRIBUTIONS

HI performed most of the experiments. YM and KW assisted in some studies. YM provided advice on measurement of melatonin metabolites using the LC‐MS/MS. AC reviewed the manuscript. AH supervised all studies and drafting of the manuscript.

## Supporting information

Fig S1

Fig S2

Fig S3

Fig S4

Table S1

Supplementary Material
