# Supplementary figures and images for: The melatonin metabolite N1‐acetyl‐5‐methoxykynuramine facilitates long‐term object memory in young and aging mice
Source: J Pineal Res. 2020 Nov 20;70(1):e12703. doi: 10.1111/jpi.12703 (PMC7816253; doi:10.1111/jpi.12703)

FIGURE S1

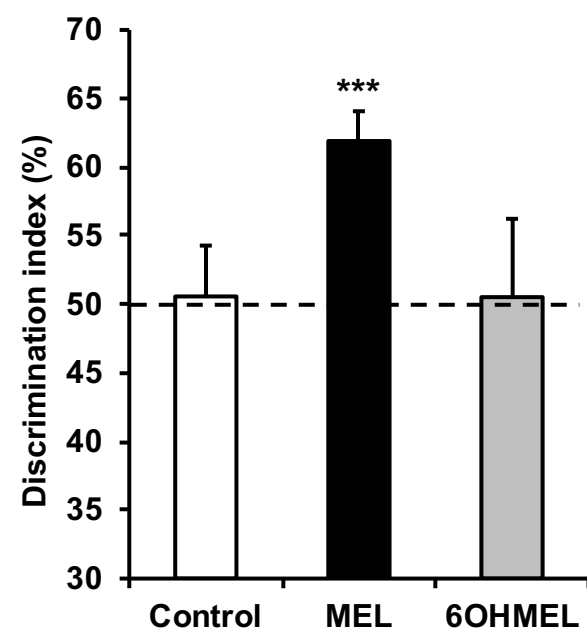

Supplement: Supplementary file 1 — Fig S1 [file JPI-70-e12703-s001.pdf]

FIGURE S2

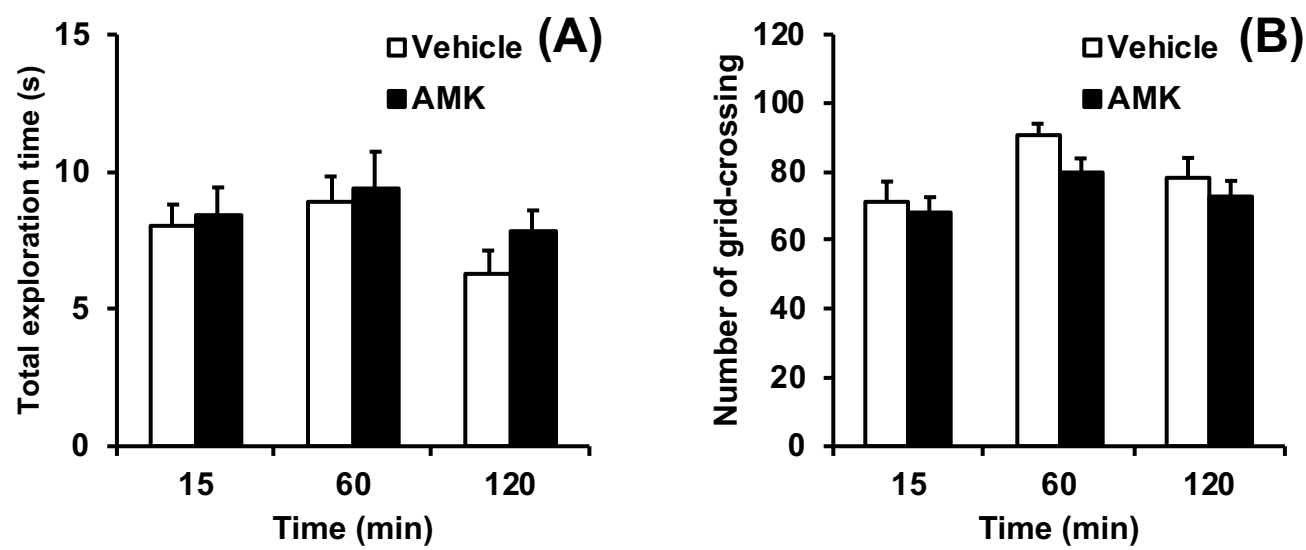

Supplement: Supplementary file 2 — Fig S2 [file JPI-70-e12703-s002.pdf]

FIGURE S3

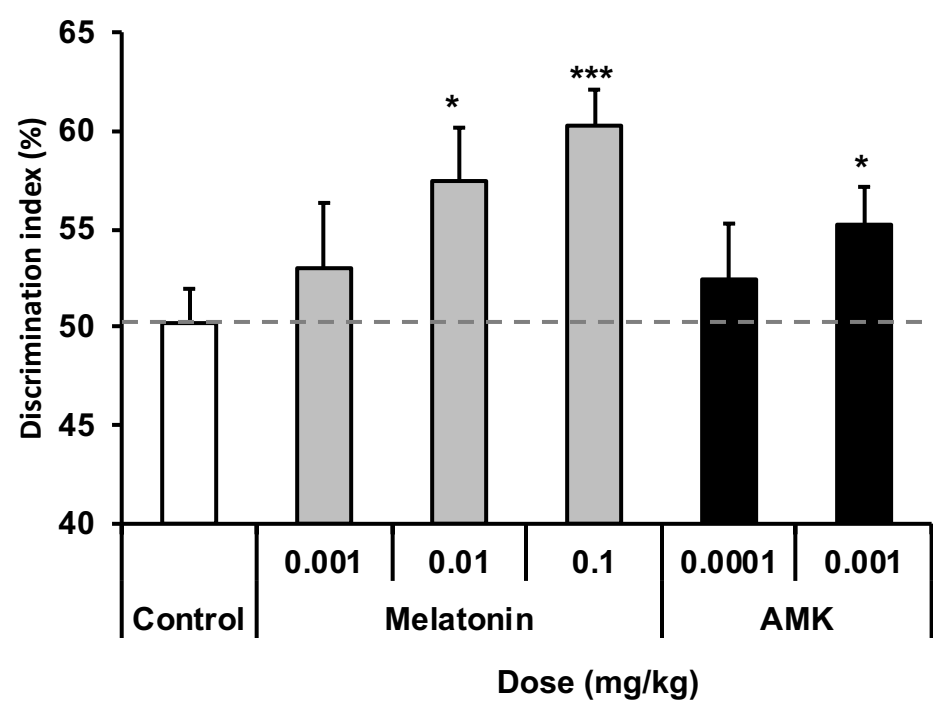

Supplement: Supplementary file 3 — Fig S3 [file JPI-70-e12703-s003.pdf]

FIGURE S4

Cont

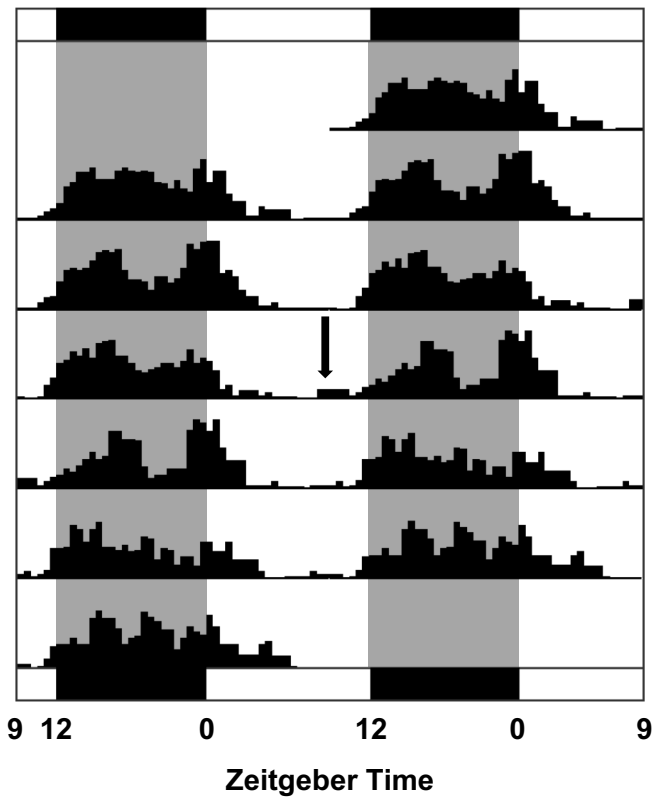

Mel

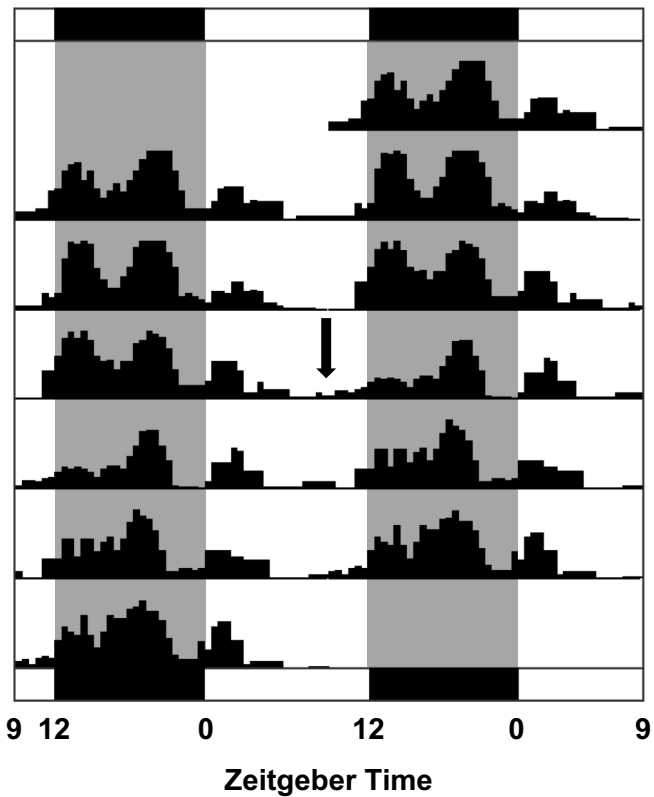

Supplement: Supplementary file 4 — Fig S4 [file JPI-70-e12703-s004.pdf]
