# Supplementary material for: The melatonin metabolite N1‐acetyl‐5‐methoxykynuramine facilitates long‐term object memory in young and aging mice
Source: J Pineal Res. 2020 Nov 20;70(1):e12703. doi: 10.1111/jpi.12703 (PMC7816253; doi:10.1111/jpi.12703)
Supplement: Supplementary file 5 — Table S1 [file JPI-70-e12703-s005.docx]

**Supplement Table**

**Table S1**: Contents of melatonin and related indoles in the pineal gland of different strain mouse

|  | **5HT (pg/gland)** | **NAS (pg/gland)** | **MEL (pg/gland)** | **AMK (pg/gland)** |
| --- | --- | --- | --- | --- |
| ICR: Day | 1410 ± 284 | 29.51 ± 5.31 | 0.19 ± 0.09 | ND |
| Night | 1302 ± 351 | 18.00 ± 3.54 | ND | ND |
| C3H: Day | 1701 ± 127 | 10.76 ± 0.46 | 45.16 ± 4.19 | 0.26 ± 0.02 |
| Night | 1716 ± 143 | 179.96 ± 14.87*** | 110.09 ± 9.37*** | 0.30 ± 0.01* |

*Note:* Values are mean ± SE from 8 animals.

**P <* 0.05, ****P* < 0.001 compared to day.
