# Supplementary material for: The melatonin metabolite N1‐acetyl‐5‐methoxykynuramine facilitates long‐term object memory in young and aging mice
Source: J Pineal Res. 2020 Nov 20;70(1):e12703. doi: 10.1111/jpi.12703 (PMC7816253; doi:10.1111/jpi.12703)
Supplement: Supplementary file 6 — Supplementary Material [file JPI-70-e12703-s006.docx]

## FIGURE S1

### Effects of 6-hydroxymelatonin on long-term object memory.

Mice were systemically administered vehicle (Control), 1.0-mg/kg 6-hydroxymelatonin (6OHMEL), or 1.0-mg/kg melatonin (MEL) 1 h following two 1-min training trials. MEL, but not 6OHMEL, significantly increased object memory at 24 h post-training. Data are presented as mean ± standard error. ****P* < 0.001 indicates significantly different than chance performance (50%). Discrimination index (%) = time exploring novel object / total object exploration time during test X 100.

## FIGURE S2

### AMK’s effects on total exploration time and grid-crossing events.

Systemic administration of 1.0-mg/kg N1-acetyl-5-methoxykynuramine (AMK) at 15, 60, or 120 min before a single 1-min training trial did not alter total exploration time (A) or number of grid-crossing events (B) compared to vehicle. Data are presented as mean ± standard error.

## FIGURE S3

### Minimally effective melatonin and AMK doses to enhance long-term object memory.

Systemic vehicle (Control), melatonin (MEL), and N1-acetyl-5-methoxykynuramine (AMK) treatment 15 min following a single 5-min training trial dose-dependently enhanced object memory at 24 h post-training. **P* < 0.05 and ****P* < 0.001 indicate significantly different than chance performance (50%). Discrimination index (%) = time exploring novel object / total object exploration time during test X 100.

**FIGURE S4**

Representative double-plotted actogram for general activity of the group administered vehicle (Cont) or melatonin (Mel).

ICR mice were housed under a 12-hour light/12-hour dark (12L12D) cycle, the same conditions used in the learning and memory tests. Melatonin (Mel) and vehicle (Cont) were also administered once at the same time point (arrows) used in the learning and memory experiment. Comparing the acrophase 3 days before and 3 days after the administration of vehicle or melatonin, the average phase delay was 26.4 ± 23.4min in the control group and 12.0 ± 21.6 min in the melatonin-administered group, with no significant difference between groups. White and black above and below each actogram depict lighting conditions and gray shading in each actogram indicates the presence of darkness.
